# PET Imaging Agents (FES, FFNP, and FDHT) for Estrogen, Androgen, and Progesterone Receptors to Improve Management of Breast and Prostate Cancers by Functional Imaging

**DOI:** 10.3390/cancers12082020

**Published:** 2020-07-23

**Authors:** John A. Katzenellenbogen

**Affiliations:** Department of Chemistry and Cancer Center at Illinois, University of Illinois at Urbana-Champaign, Urbana, IL 61801, USA; jkatzene@illinois.edu, Tel.: +1-217-333-6310; Fax: +1-217-333-7325

**Keywords:** FES, FFNP, FDHT, receptor-targeting, PET imaging, radiopharmaceuticals, endocrine therapy, breast cancer, prostate cancer, hormone-challenge test

## Abstract

Many breast and prostate cancers are driven by the action of steroid hormones on their cognate receptors in primary tumors and in metastases, and endocrine therapies that inhibit hormone production or block the action of these receptors provide clinical benefit to many but not all of these cancer patients. Because it is difficult to predict which individuals will be helped by endocrine therapies and which will not, positron emission tomography (PET) imaging of estrogen receptor (ER) and progesterone receptor (PgR) in breast cancer, and androgen receptor (AR) in prostate cancer can provide useful, often functional, information on the likelihood of endocrine therapy response in individual patients. This review covers our development of three PET imaging agents, 16α-[^18^F]fluoroestradiol (FES) for ER, 21-[^18^F]fluoro-furanyl-nor-progesterone (FFNP) for PgR, and 16β-[^18^F]fluoro-5α-dihydrotestosterone (FDHT) for AR, and the evolution of their clinical use. For these agents, the pathway from concept through development tracks with an emerging understanding of critical performance criteria that is needed for successful PET imaging of these low-abundance receptor targets. Progress in the ongoing evaluation of what they can add to the clinical management of breast and prostate cancers reflects our increased understanding of these diseases and of optimal strategies for predicting the success of clinical endocrine therapies.

## 1. Introduction

**Figure 1:**
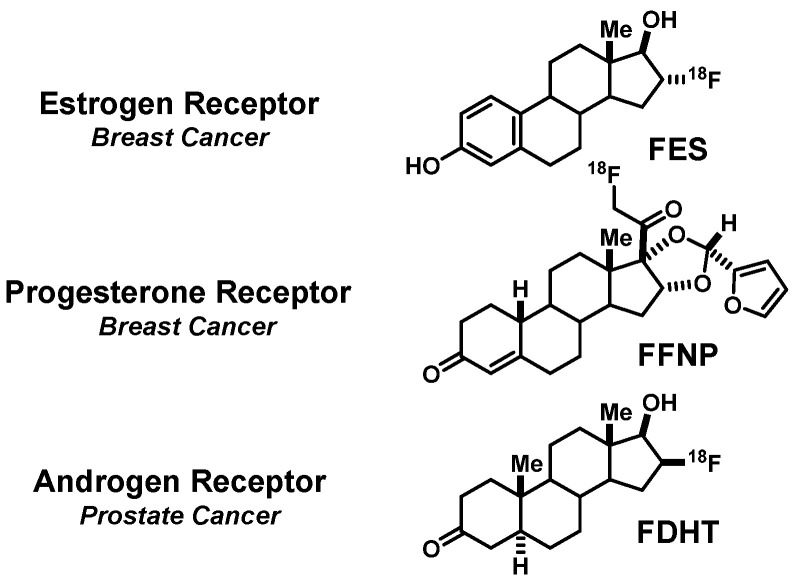
Structures of 16α-[^18^F]fluoroestradiol (FES), 21-[^18^F]fluoro-furanyl-nor-progesterone (FFNP) and 16β-[^18^F]fluoro-5α-dihydrotestosterone (FDHT), with their hormone receptor targets and use in cancer noted at left.

Steroid receptors are transcription factors that play major roles during development, and in both adult men and women, they control reproduction and regulate many other organ systems; they are also important regulators of the growth and spread of various hormone-dependent cancers. This review will cover principally the development of agents to image the estrogen receptor (ER) and the progesterone receptor (PgR) in breast cancer and the androgen receptor (AR) in prostate cancer. Imaging agents for these targets have been labeled with different radioisotopes (various radiohalogens, carbon-11, and radiometals) and have been developed for use with different tomographic imaging devices. Here, I will cover the development of three positron emission tomography (PET) imaging agents labeled with fluorine-18 that have reached the clinic and continue to be of great utility and actively investigated: 16α-[^18^F]fluoroestradiol (FES) and 21-[^18^F]fluoro-furanyl-nor-progesterone (FFNP) for breast cancer and 16β-[^18^F]fluoro-5α-dihydrotestosterone (FDHT) for prostate cancer (Figure 1).

The initial synthesis, radiolabeling, and in vivo studies in animal models and humans for these three PET probes were initiated in the 1970s and 1980s, and culminated with first-in-man/woman reports for FES in 1984 [1,2], FDHT in 2005 [3], and FFNP in 2012 [4]. For several prior years, early investigations leading to the development of these agents animated the frontier of the rapidly evolving field of receptor-binding radiotracers. The results from our research and that of others, and the insights we all gained from them, were often taking the lead in defining the technical advances and conceptual understanding that are now common knowledge in the design and development of radiotracers for limited capacity high affinity receptor targets. The technical advances were in isotope production; chemical reactivity of the radionuclide; and efficiency, ease, and speed of radiolabeling and product purification; the conceptual understandings were of performance criteria critical for receptor-targeted radiotracers, i.e., target binding affinity; selectivity and capacity; imaging probe molar activity; and distribution, metabolism, and clearance behavior. Attention to all of these factors was needed to produce PET probes that gave informative in vivo images, because only low levels of these high affinity receptors are present in human breast and prostate tumors. In the three sections that follow, these facets of technical and conceptual advances are wrapped into historical accounts of the development of FES, FDHT, and FFNP, including informative missteps that occurred along the development pathways. Each of these three sections ends with a description of efforts to improve these three clinically used PET imaging agents, including some suggestions for future studies that might be worthwhile. The final section provides a summary of clinical investigations involving these three agents, with an indication that many new studies are ongoing and others still emerging. While discussion, here, centers on the use of these PET probes in breast and prostate cancers, in principle, they might also be useful in other types of cancers that are regulated by hormone action through ER, PgR, or AR.

**Figure 2:**
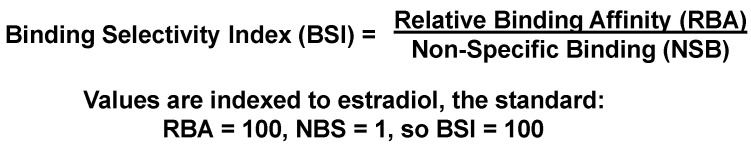
Definitions of relative binding affinity (RBA), nonspecific binding (NSB), and binding selectivity index (BSI).

Throughout the sections on probe development, we will make frequent use of several terms to characterize the binding behavior of receptor-targeted radiotracers; hence, these deserve some explanation (Figure 2).

- **RBA** and **K_i_**: Affinity for the intended target is expressed relative to a standard ligand, typically a tritiated version of a known high-affinity ligand used as a tracer in convenient radiometric competitive binding assays, such as [^3^H]estradiol for ER [5]. Thus, for ER, the standard, estradiol (which has a K_d_ value of 0.2 nM), is assigned a relative binding affinity (**RBA**) value of 100, with lower affinity candidate tracers having RBA values below 100, and higher affinity ones above 100. If of interest, K_i_ values can be calculated from RBA values: K_i_ (in nM) = (0.2 × 100)/RBA. The standard compound for PgR is R5020, with a K_d_ of 0.4 nM [6,7], and for AR is methyltrienolone (R1881), with a K_d_ of 0.2 nM [6].
- **NSB** and **BSI:** Nonspecific binding (**NSB**) is high-capacity, low-affinity, off-target binding, to proteins such as serum albumin and possibly lipid phases, and it is also measured relative to that of the standard ligand, but in this case, the standard is assigned an NSB value of 1. As a measure of target binding selectivity, we defined the ratio of specific to nonspecific binding as the binding selectivity index, **BSI**: BSI = RBA/NSB [8,9]. Thus, the standard ligand has a BSI of 100 (i.e., 100/1), and candidate tracers with higher selectivity have BSI values above 100, whereas those with lower selectivity have BSI values below 100. Notably, unlike RBA, BSI is sensitive to both target binding affinity and nonspecific binding.

NSB can be measured experimentally by various, though somewhat tedious, methods such as equilibrium dialysis with serum or serum albumin [9]. We and others have found that NSB is proportional to compound lipophilicity, which is typically expressed as an octanol–water partition coefficient, Log*P_o/w_*, and can be measured more conveniently by octanol–water shake flash methods [7] or by reversed-phase HPLC after suitable calibration [10]. Even more conveniently and of equal accuracy, NSB values can be based on calculated Log*P_o/w_* values [9].

## 2. FES, a PET Imaging Agent for Estrogen Receptors in Breast Cancers

### 2.1. A Prelude to In Vivo Imaging of ER: Finding a Ligand (Steroidal vs. Nonsteroidal) and a Radionuclide (Iodine, Bromine, or Fluorine) Enabling Effective and Selective Uptake by ER-Target Tissues

**Figure 3:**
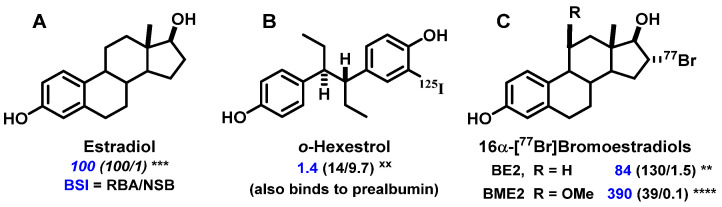
Estrogen receptor ligands. (**A**) Estradiol, the endogenous estrogenic hormone, (**B**) *o*-Iodohexestrol, (**C**) bromoestradiols, 16α-[^77^Br]bromoestradiol (BE2) and 16α-[^77^Br]bromo-11β-methoxyestradiol (BME2). Under each compound, the numbers indicate the binding selectivity index (BSI), which is the ratio of the relative binding affinity (RBA) to the nonspecific binding (NSB); all three values are scaled relative to estradiol as the standard, set at 100. The number of asterisks indicate the degree of uptake efficiency and selectivity in the uterus of immature rats. ^XX^ indicates no estrogen-specific uterine uptake.

Our earliest interest in preparing gamma-emitting estrogens was not at all for the purpose of in vivo imaging, but rather to study the kinetics of estrogen uptake in ER-positive tissues, followed by the exchange of estrogens once bound by the receptor. We planned to do this by preparing iodine-125 and iodine-131 labeled hexestrol, a nonsteroidal ligand for ER. Hexestrol has very high affinity for ER (RBA = 300; K_d_ = 0.07 nM), and while progressive iodine substitution reduced binding affinity, monoiodohexestrol having a single iodine adjacent to one phenol still bound quite well to ER (RBA 14; K_d_ 1.4 nM) [11,12] (Figure 3). It was fortuitous that early in 1974, when I presented early phases of this work at a meeting of the American Chemical Society in Los Angeles, I learned of the possibility of using such compounds for in vivo imaging of ER-positive breast cancer by gamma scintigraphy. Shortly thereafter, we performed our first biodistribution study of [^125^I]iodohexestrol in immature female rats; immature animals were used because their endogenous levels of estradiol are low and would not compete with probe binding to ER. In addition, it was well known that high molar activity [^3^H]estradiol was taken up avidly and selectivity by the uterus [13,14]. The results with [^125^I]iodohexestrol, however, were disappointing, but instructive: unlike [^3^H]estradiol, uterine uptake of [^125^I]iodohexestrol was only modest and was not blocked by unlabeled estradiol [12] (Figure 3).

Initially puzzled by these results, we took a deeper look at the binding characteristics of [^125^I]iodohexestrol to consider not only ER binding affinity but also nonspecific binding, NSB, i.e., binding to abundant low-affinity components such as serum albumin and lipid phases. In doing this, we articulated the concept of the binding selectivity index, BSI, which accounts of both the desired high affinity target binding and the undesired nonspecific, off-target binding (see Figure 2 and Figure 3) [9]. Estradiol by definition had a BSI value of 100 (RBA of 100/NSB of 1), but by this metric, iodohexestrol, with both a lower RBA of 14 and a much higher NSB of 10, had it an embarrassingly low BSI of 1.4 [12]. Clearly, the very low BSI value for [^125^I]iodohexestrol provided some explanation for its poor biodistribution. Later, however, we found another reason: the structure of iodohexestrol sufficiently resembles the terminus of thyroid hormone that it is tightly bound by transthyrethrin, a thyroxine-binding prealbumin that is an abundant in serum protein. This off-target interaction overshadows any hint of the uptake of iodohexestrol by the high affinity but limited capacity of ER in the uterus. The off-target binding of iodohexestrol to a serum protein also foreshadowed marked effects we later found with another serum binding protein, sex-hormone binding globulin (SHBG), a protein thought to facilitate the in vivo distribution of certain steroids. As will be noted later, binding to SHBG can have marked effects, both good and bad, on the biodistribution of ER and AR-targeted PET radiotracers that merits further investigation [15,16,17,18].)

Fortuitously, later in 1974, I met and joined forces with Michael Welch a Professor of Radiological Sciences at Washington University Medical School in St. Louis, about a 3-h drive from the University of Illinois at Urbana-Champaign; this began a fruitful collaboration in radiopharmaceutical development that extended for nearly 38 years. At the time, other researchers interested in imaging ER in breast cancers were seeing favorable biodistribution using steroidal estrogens radioiodinated at different positions [19,20,21,22,23,24,25]. At the time, Dr. Welch was receiving bromine-77 shipments from Los Alamos National Laboratory for other purposes (protein labeling); so, we began to prepare bromoestrogens. In general, 16α-bromoestradiol (BE2), readily prepared by electrophilic bromination of a 17-enol acetate precursor, bound to ER with greater affinity than estradiol (Figure 3) [26], and BE2 and other analogs could be radiobrominated efficiently on a tracer scale [27], giving products with very high molar activities [28,29]. Biodistribution studies confirmed that these analogs were taken up efficiently and selectively by the uterus of immature female rats, now nicely mimicking the selective uptake of [^3^H]estradiol [27]. In an adult rat, BE2 was also taken up selectively by ERα-positive DMBA (dimethylbenzanthracene)-induced mammary tumors [27].

The lesson we learned from iodohexestrol that both high receptor affinity and low nonspecific binding were important was confirmed by the behavior of these radiobrominated estrogens, because their BSI values (shown in Figure 3) were more useful predictors of their in vivo uptake efficiency and selectivity than their binding affinity (RBA) values alone, particularly in predicting the retention of a tracer by the uterus [30]. Compared to the parent bromoestrogen, 16α-[^77^Br]bromoestradiol (BE2), with a BSI of 84 (130/1.5) was similar to estradiol; the more polar 16α-[^77^Br]bromo-11β-methoxyestradiol (BME2) actually bound to ER 4-fold less well (RBA 39) but had a 10-fold lower NSB (0.1), giving it a much higher BSI value of 390 (39/0.1). Indeed, the uptake of BME2 by the uterus was greater than that of BE2. Its target tissue retention, in particular, was much longer (t_1/2_ of 6 h for BE2 vs. 24 h for BME2) [28], clearly showing evidence for the pronounced target-tissue retention that can be displayed by compounds having high BSI values [30,31].

These radiobrominated estrogens were simple to prepare, had excellent binding characteristics, and gave very efficient and ER-selective biodistributions. Our attempts to use them for in vivo imaging with 2D gamma cameras, however, was confounded by the high emission energy of bromine-77. Some accumulation of [^77^Br]BE2 activity above a relatively high background could be noted in DMBA-induced mammary tumors in rats [26,27], but in humans, the uptake of [^77^Br]BE2 in ER-positive breast tumors was obscured by extensive scattering from other sites [32]. This prompted us to switch isotopes to fluorine-18 to benefit from its well-recognized advantages of an element that is small and forms very strong bonds to carbon. While its high electronegativity gives the C-F bond a large dipole moment, the potent estrogen, estriol, also has a polar OH group at the 16α position and binds well to ER.

Our switch to a positron-emitting radionuclide fit well with the rapid development of PET imaging technology at Washington University, but at this early time, there were challenges both in the cyclotron production of fluorine-18 and its use for radiolabeling. Fluorine-18 was being produced in gas targets (^20^Ne(d,α)^18^F) and was used to prepare 2-[^18^F]fluoro-2-deoxyglucose by an electrophilic reaction using [^18^F]F_2_. Efficient extraction of activity from the gas target required carrier F_2_, which reduced molar activity far below what is useful for receptor imaging. For nucleophilic radiofluorination, which we planned to use, efficient extraction of the activity from gas targets without dilution (e.g., as H [^18^F]F) proved to be very cumbersome [33]. Soon thereafter, fluorine-18 production in oxygen-18 water targets (^18^O(p,n)^18^F) gave large quantities of [^18^F]fluoride ion that was easily recovered from the target as an aqueous solution. Rapid drying by azeotropic distillation with acetonitrile in the presence of lipophilic cations or cation chelators produced [^18^F]fluorine in an organic soluble, chemically reactive form [1,2,34]. A final technical hurdle required us to trace the source of fluorine-19 that was inadvertently and markedly reducing the specific activity of our fluorine-18 radiolabeled products [2]. The source was Teflon tubing used for activity transfers from the target and during synthesis that released fluoride-19 ion when exposed to hard radiation. Replacement with polyethylene tubing provided us with the very high molar activities required for in vivo imaging of low abundance targets such as ER [2]. A more extensive study of fluoride ion release from Teflon lines during radiochemical syntheses has recently been published [35].

### 2.2. An Exploration of Various [^18^F]Estrogens and the Birth of FES

**Figure 4:**
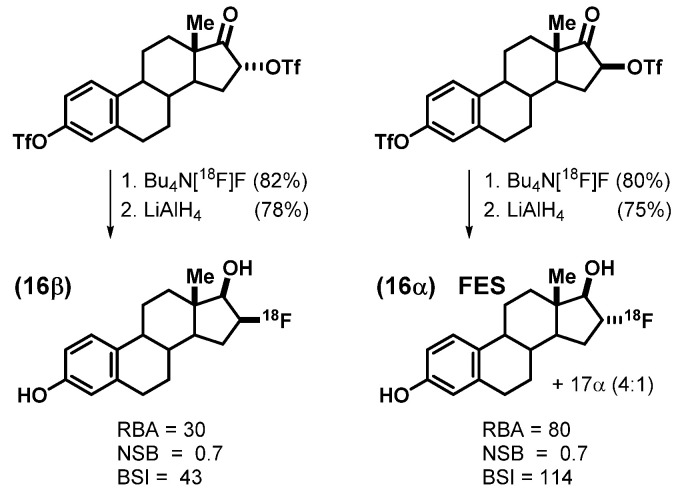
Synthesis of the two 16-[^18^F]fluoroestradiols and their binding values. FES (the 16α-epimer, bottom right) was carried forward for positron emission tomography (PET) imaging studies.

We prepared a number of fluorine-labeled steroidal and nonsteroidal estrogens [1,36,37,38], but soon focused on the 16 epimers of fluoroestradiol, which were prepared by [^18^F]fluoride ion displacement of the corresponding epimeric 16 trifloxy estrones, which proceeded with inversion of stereochemistry; the second triflate protected the 3-phenol (Figure 4). Subsequent reduction of the 17-ketone was done with LiAlH_4_, first at low temperature (−78 °C) to give the 17β-epimeric alcohol with good selectivity and then warming to RT to cleave the 3-O-triflate [1]. The 16α epimer of the product bound twice as well to ER than did the 16β isomer, giving BSI values of 114 (80/0.7) for 16α and 43 (30/0.7) for 16β [39]. Thus, 16α-[^18^F]fluoroestradiol, which we named, FES, became our tracer of interest. We could routinely obtain FES with molar activities well in excess of 1000 Ci/mmol (37 GBq/μmol) [2], which were sufficient for in vivo PET imaging [40]. Following our original publication of the synthesis of FES [2], alternative routes have been reported, some of which avoid stereochemical issues at C-17 [41,42,43] and are more adaptable to automated synthesis systems [44,45,46], which were not available when we first developed our synthesis.

**Figure 5:**
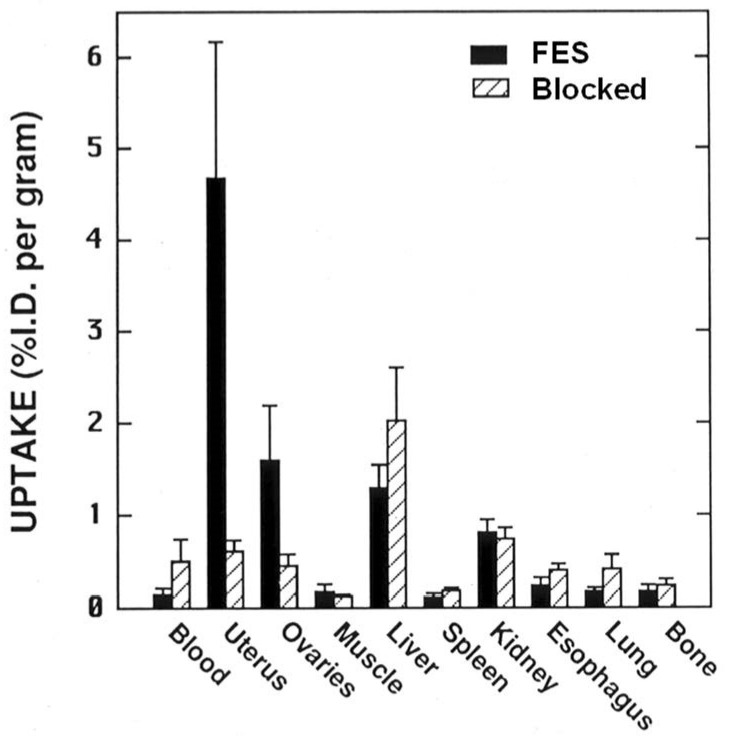
Tissue biodistribution of FES at 1 h in immature rats alone or blocked with unlabeled estradiol. (Prepared from data in reference [2].)

In biodistribution studies, FES showed high uptake in the uterus and ovary of immature female rats that was nearly completely reversed by a blocking dose of unlabeled E2 (Figure 5). Uptake in nontarget organs varied, but was unchanged by the blocking dose of E2. Defluorination was minimal, noted by the absence of uptake in bone. Other F-18 labeled estrogens we investigated around the same time showed no particular advantage over FES [2]; so, FES, on which we published first in 1984 [2], became the focus of our clinical development efforts, all done in collaboration with Washington University colleagues.

It is worth noting that in subsequent years, we did undertake a substantial effort to improve upon FES in terms of its binding selectivity and its target uptake efficiency and selectivity by introducing various substituents at the 11β and 17α positions. These studies are described in detail elsewhere [47], but in essence, although we were able to obtain compounds that had better RBA and BSI binding values and were substantially to markedly better than FES in terms of their biodistribution in rodents, they were not better in humans [18]. This unexpected species difference appears to involve differences in the binding characteristics of serum steroid binding proteins in rodents (α-fetoprotein) vs. humans (sex hormone binding globulin). It is an issue that is still not well understood, and also appears to affect the behavior of the PET imaging agents for the androgen receptor (see Section 3). More recently, versions of FES in which strategically placed substituents, including unlabeled fluorine, appear to enhance binding characteristics and reduce metabolic clearance, are reported to provide somewhat better images than those obtained with FES [48].

In addition to the preclinical toxicology studies required at the time for the use of high molar activity radiotracers in humans, we examined the behavior of FES in rodent models of breast cancer. In DMBA-induced mammary tumors in adult female rats, we examined the time course of FES tumor uptake and metabolism and looked for correlations between metrics of blood volume and blood flow in tumors with ER levels in tumors and in the uterus; these relationships proved to be complex and difficult to interpret [49]. On the other hand, a dose–response study of FES in immature female rats was very illuminating. The uptake of FES in ER-rich tissues (uterus and ovary) became saturated only at the higher mass dose levels, whereas tissues with lower ER levels, such as muscle and esophagus, showed low but clearly measurable levels of specific uptake of FES activity that was saturated at much lower mass dose levels [50]. To us, this indicated that while the uptake of FES in very ER-rich target organs might be “flow limited” and not proportional to high ER levels [31], the specific uptake by the lower levels of ER found in breast tumors were likely to be proportional to receptor levels [40].

**Figure 6:**
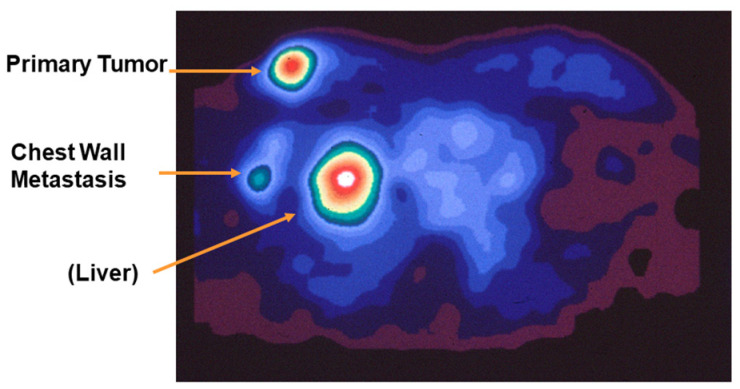
FES-PET of a breast tumor in a woman with breast cancer. Image of the Year at the 1987 National Meeting of the Society of Nuclear Medicine. (Image provided by Drs. Michael Welch and Barry Siegel, Washington University Medical School; discussed (but not shown) in reference [52].)

In 1988, we published the first PET images of ER-positive breast tumors in women [51], and one image, presented in advance of our publication at the 1987 national meeting of the Society of Nuclear Medicine, was highlighted by Professor Henry Wagner as “Image of the Year” (Figure 6) [52]. Because it was the first PET image of a receptor in cancer, it represented a milestone in the development of receptor-targeted radiotracers for PET imaging. A discussion of the clinical utility of FES-PET imaging ER in breast cancer, as well as that for FDHT-PET and FFNP-PET, is given in Section 5.

The focus of this review on PET imaging of steroid receptors in cancer, using radiofluorinated probes, does not provide an adequate opportunity to cover other ER probe development and clinical imaging work using radioiodinated steroids. Some of these have very good ER binding affinity, showed promise in biodistribution studies in rodents [20] and proceeded into interesting clinical studies [53,54]. Details can be found in the references cited and [55]. The biodistribution of some radioiodinated nonsteroidal estrogens also looks very promising [56], and some work has been done to advance these and related agents for both imaging and targeted in vivo radiotherapy in preclinical breast cancer models [57,58]. Note is made of other recent reviews covering the development of PET imaging agents for ER [47,59].

## 3. FFNP, a PET Imaging Agent for Progesterone Receptors in Breast Cancers

### 3.1. Bumps on the Road to the Discovery of a PET Radiotracer for PgR in Breast Cancer

Early reports of PET imaging agents for the progesterone receptor (PgR), published in the 1970s and 1980s, described the synthesis of 21-[^18^F]fluoroprogesterone, a direct analog of the endogenous progestational hormone, progesterone, as well as some analogs of this compound and ones substituted at different sites; however, no PgR binding was reported, and biodistribution and metabolism studies were limited and generally disappointing [60,61,62]. High-affinity radioiodinated ligands for PgR, reported at the same time, appeared more promising, especially (Z)-17β-hydroxy-17α-(2-iodovinyl)-4-estren-3-one and the bromo analog [63,64,65,66,67], and these showed some evidence of PgR-specific uptake in the uterus of estrogen-primed immature rats [24,68].

**Figure 7:**
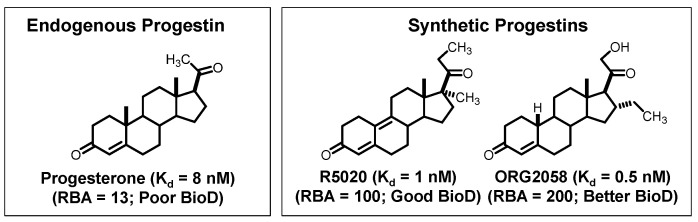
Natural and synthetic ligands for the progesterone receptor (PgR). Binding affinities and biodistribution (BioD) properties.

We began our development of PgR PET probes knowing that progesterone itself is not a very high-affinity ligand for PgR (K_d_ = 8 nM) [6,69]. Hence, we focused on two much higher affinity ligands that had been developed by pharmaceutical companies, the Roussel compound R5020 (K_d_ = 1 nM) [70] and the Organon compound ORG2058 (K_d_ = 0.5 nM) [71] (Figure 7). As will be noted below, to some degree, structural changes that boost the binding affinity of androgens for AR also worked for the progestins: ORG2058 is a 19-nor analog of progesterone with an additional 16α-ethyl group, and R5020 has an extended A,B-ring dienone with flanking methyl groups at the 17α and 21 positions [6,69].

Because these high-affinity PgR ligands were widely used as radiotracers in competitive binding assays, they were available in tritium-labeled form. R5020, the better-known and generally favored tracer, was assigned a relative binding affinity (RBA) of 100, which gave ORG2058 a higher RBA of 200. In biodistribution studies, both tritium-labeled progestins showed high, specific (i.e., blockable) uptake in the uterus of immature rats that had been primed with estradiol to increase the levels of uterine PgR [72]. Notably, due to its low binding affinity, tritium-labeled progesterone showed poor PgR-selective uptake by the uterus [72].

**Figure 8:**
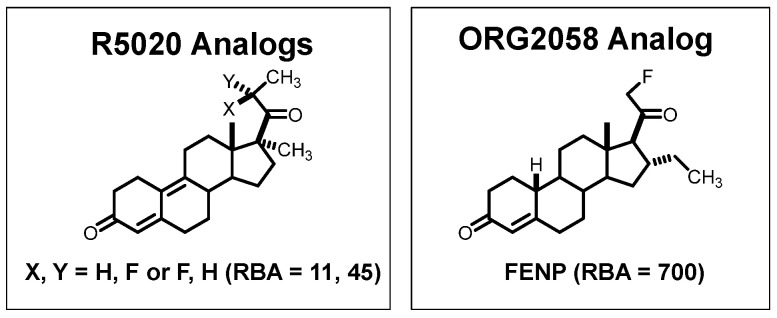
Fluorine-18 labeled analogs of the high affinity PgR ligands, R5020, and ORG2058. Binding affinities are relative to R5020, set at 100.

The 21-hydroxy group in ORG2058 was an obvious site for fluorine substitution and had already been explored by researchers at Organon [73]. Fluorine-18 labeling by fluoride ion displacement of the corresponding triflate gave 21-fluoro-16α-ethyl-nor-progesterone (FENP) (Figure 8), which bound to PgR 3-times better than ORG2058 and 60-times better than progesterone, giving it a remarkably high binding affinity (RBA 700; K_i_ = 0.14 nM) [74]. While there was no such obvious site for fluorine substitution in R5020, two epimeric 21-hydroxy metabolites of R5020 were known and still had reasonably good binding affinities [75]. Fluorine for hydroxyl substitution at these sites gave epimeric products having more modest binding affinities (RBA values of 11 and 45) (Figure 8) [75]. All three of these products showed efficient and specific uptake in the uterus of estrogen-primed rats. Not surprisingly, however, the uptake of the very high affinity FENP was clearly the most efficient and selective [74,75].

**Figure 9:**
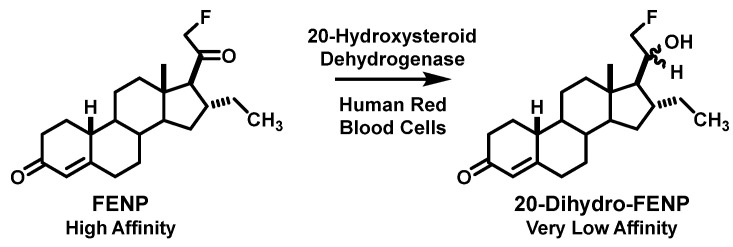
Rapid metabolic inactivation of FENP in humans (but not in rats), presumed due to a very active 20-hydroxysteroid dehydrogenase in human blood, but absent from rodents.

Encouraged by these promising preclinical studies, we tried to use FENP to image ER and PgR-positive breast tumors in women and were most surprised to find that there was little tumor uptake at all [76]. At the same time, a group in the Netherlands, who were also investigating FENP and related compounds for PgR imaging, had the same experience [77,78]. When the Netherlands group examined FENP metabolism in humans, they found that a very active 20-hydroxysteroid hydrogenase rapidly converts circulating FFNP to the low affinity 20-hydroxy metabolite, presumed to be the 20α epimer (Figure 9) [79]. In humans, this active dehydrogenase appears to be in blood cells, but curiously, it is not present in rodents and thus, did not undermine the very favorable biodistribution of FENP in our preclinical models [77,80]. Clearly, this marked and unexpected species difference in steroid metabolism made FENP unsuitable for imaging breast cancer in humans [76]. In some ways, the finding of compounds that were promising in rodent models proved most unsuitable for imaging in humans was reminiscent of our experience with the behavior of certain modified FES [18] and FDHT analogs [17], whereas in those cases for a different reason: lack of sufficient binding affinity for SHBG.

### 3.2. Defeating a Metabolic Inactivation in Human Blood Led to the Discovery of FFNP as an Effective PET Imaging Agent for PgR in Breast Cancer

**Figure 10:**
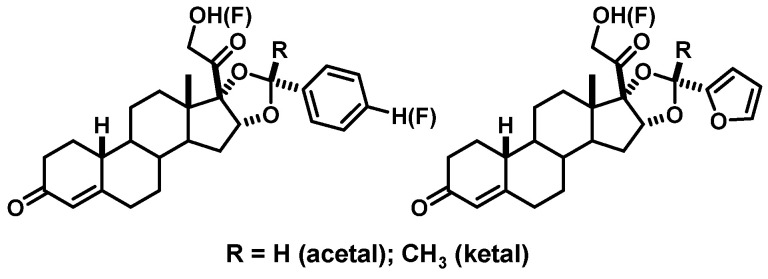
Compound 19-norprogestins with 16α,17α-dioxolanes from benzaldehyde/acetophenone (left) and furfural/furanyl methyl ketone (right). The steric bulk near the C = O group was intended to block its metabolic inactivation by 20-hydroxysteroid dehydrogenase action.

From a number of early medicinal chemistry studies, we were aware that PgR was very tolerant of large substituents on the alpha face of the D-ring of ligands, and we found that researchers at Squibb in the 1960s had developed potent progestins having acetals and ketals appended to 16α,17α-dihydroxy progestins (Figure 10) [81,82]. We thought that these bulky substituents, situated directly adjacent to the metabolically vulnerable 20-keto group of progestins, might sterically impede their inactivation by the 20-hydroxysteroid dehydrogenase. Therefore, we undertook a systemic investigation of fluoro-19-norprogestins having various 16α,17α-dioxolane substituents (initially to make affinity labels for PgR) [83]. In one series, the fluorine substitution was on the para position of dioxolanes from benzaldehyde or acetophenone, with radiofluorination being possible by nucleophilic aromatic substitution on the corresponding *p*-nitro compound prior to dioxolane formation [7]. Alternatively, fluorine substitution at C21 could be done as a last step on the 21-triflate prepared from preformed dioxolanes of unsubstituted benzaldehyde or acetophenone. Some of these compounds were quite lipophilic, and their biodistribution selectivity was not very high [7]. To reduce lipophilicity and nonspecific binding, we turned to dioxolanes prepared from more polar arene carbonyl compounds, notably furfural or acetylfuran [80]. With all of these dioxolanes, the arene substituent can either be endo (folded under the D-ring) or exo (extended outward from the D-ring); these epimers could be well separated by chromatography, and the endo stereoisomer had much higher affinity [7,80].

**Figure 11:**
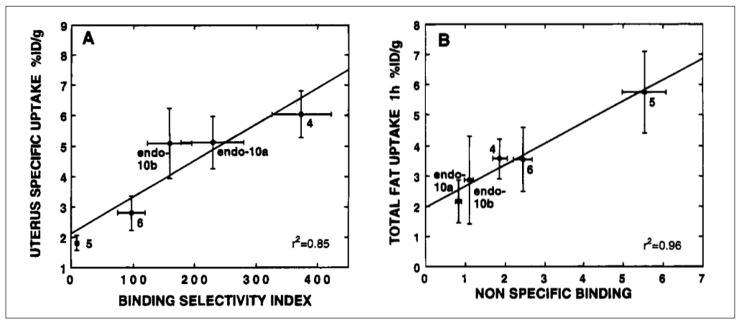
Correlations between (**A**) binding selectivity index (BSI) and PgR-specific uterine uptake, and (**B**) nonspecific binding (NSB) and uptake into fat for five different PgR imaging agents. (Adapted and reprinted with permission from reference [80]. Copyright 1995, American Chemical Society.)

Based on the PgR binding affinities and calculated NSB values of the fluorine-19 substituted 16α,17α-dioxolanes, we selected a number for fluorine-18 radiolabeling and in vivo biodistribution studies. All of them gave clear, blockable uptake in the uterus of our adult estrogen-primed rat preclinical model [80]. Their target tissue uptake selectivity (uterus/muscle and uterus/blood ratios) reflected both their PgR binding affinities and their lipophilicities. In fact, the correlation between the target/nontarget uptake ratios and the binding selectivity index (BSI) for these compounds was much better than with just their RBA values; uptake in fat also correlated nicely with their NSB values (Figure 11) [80]. This highlights the value of considering target-specific vs. nonspecific binding affinity, embodied in the BSI value, as a useful predictor of in vivo uptake in target tissues (see Section 1 and Section 2.1) [40].

**Figure 12:**
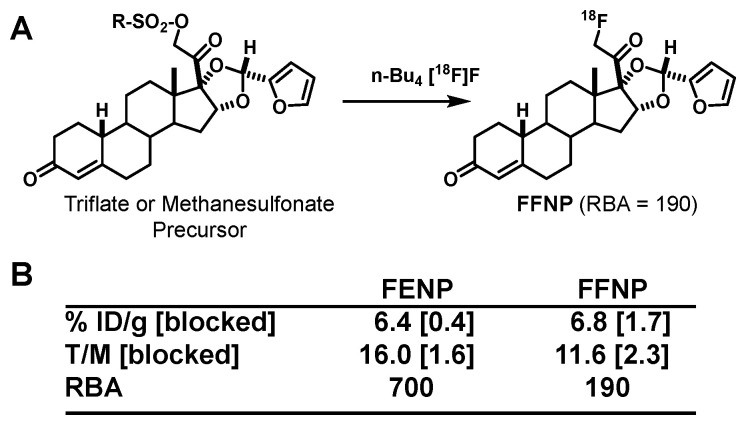
(**A**) Synthesis of FFNP, 21-[^18^F]fluoro-furanyl-nor-progesterone. (**B**) Comparison of the binding and biodistribution properties of FFNP vs. FENP.

The results from these extensive biodistribution studies indicated that the more hydrophilic acetals from furfural had the most favorable combination of high PgR-mediated target tissue uptake and low nonspecific binding, the endo isomer being the best, as expected. For convenience, we named this compound FFNP (for 21-[^18^F]Fluoro-16α,17α-endo-Furanyl-19-Nor-Progesterone) (Figure 12A). In more recent preclinical imaging studies in mice, FFNP showed efficient and selective PgR-mediated uptake in a syngeneic mouse mammary cancer system [84,85]. Clinical studies with FFNP in breast cancer are discussed in Section 5.2. Clinical studies with FFNP will likely be facilitated by an expedited radiosynthetic method reported recently [86]. An interesting comparison can be made between the PgR binding affinities and biodistribution properties of FFNP and FENP (Figure 12B): FENP has nearly fourfold higher binding affinity, yet the uptake of FFNP by the uterus of estrogen-primed immature rats was as good as that for FENP in terms of %ID/g and nearly as good in terms of tissue to muscle ratio (T/M) and blocking by a high dose of unlabeled compound; of course, irrespective of its high PgR binding affinity, FENP failed rather dramatically for clinical PgR PET imaging in vivo, because of its rapid metabolic inactivation by the 20-hydroxysteroid dehydrogenase activity found in humans (Figure 9).

### 3.3. Alternative Approaches and Efforts to Improve PET Imaging Agents for PgR

**Figure 13:**
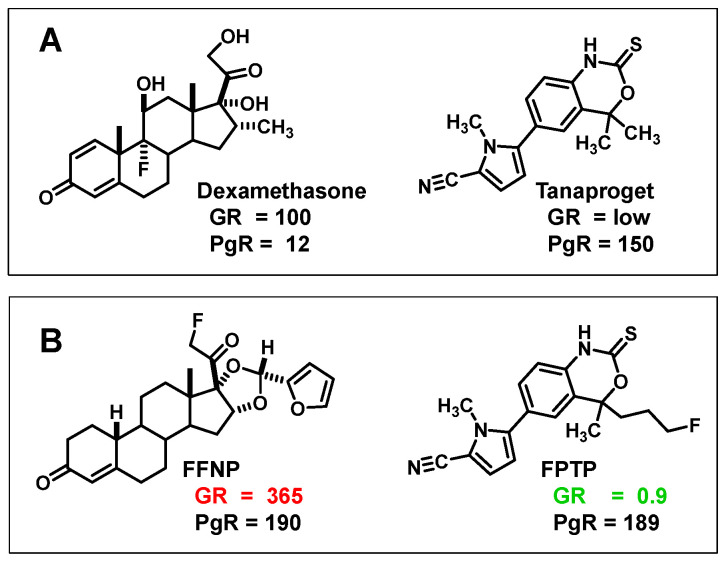
(**A**) Nonsteroidal PgR ligand, tanaproget, compared to glucocorticoid receptor (GR) ligand dexamethasone. (**B**) Comparison of FFNP and FPTP RBA to GR vs. PgR.

While FFNP has very good binding affinity for PgR, it also binds well to the glucocorticoid receptor (GR) [87]. GR is a challenging target for PET imaging [88]; however, because there are significant levels of GR in many breast cancers [89], we had concerns that GR binding by FFNP might confound PET imaging of PgR in breast cancer. Assurances that this might not be a problem came from preclinical studies in which we found that FFNP uptake in syngeneic murine mammary tumors could be efficiently blocked by coadministration of a blocking dose of the specific PgR ligand, R5020, but not by the specific GR ligand, dexamethasone [84]. Nevertheless, we were curious whether we might be able to avoid any issues with off-target GR binding by developing a PgR radiotracer having very low affinity for GR, by basing them on tanaproget, a nonsteroidal ligand with high PgR but very low GR binding affinity (Figure 13A) [90].

We prepared a number of fluorine-substituted tanaproget analogs with high PgR and low GR binding affinities [91]; we prepared one of them, fluoropropyl tanaproget, FPTP (PgR RBA 189 and GR RBA 0.9 [dexamethasone, standard, RBA 100]) (Figure 13B), in fluorine-18 labeled form [87]. FPTP showed excellent uptake in the uterus of estrogen-primed rats that was PgR-mediated and equivalent to that of FENP and FFNP, and its activity in bone was considerably less than that of these two [87]. Based on its structure, FPTP should be unaffected by the 20-hydroxysteroid hydrogenase activity in humans that undermined the utility of FENP [79]. So, FPTP is an obvious candidate for clinical translation, but given that FFNP was already being advanced into clinical studies by our groups (NCT02455453) [4], we made no further efforts to develop FPTP. Other fluorine-18 tanaproget derivatives have been investigated, but showed limited promise for imaging PgR in vivo [92,93]. Hence, at this point, FFNP is the most advanced PET imaging agent for PgR in breast cancer patients, where it appears to have good selectivity for PgR (see Section 5.2).

Some iodine and bromine-substituted analogs of 16α,17α-dihydroxy norprogesterone acetophenone dioxolanes were prepared [94]. They have good PgR binding affinity and show efficient and selective PgR-mediated uptake in vivo; with alternative radionuclides (iodine-123 and bromine-76/77), they might be useful for radiotherapeutic applications [94]. Note is made of another recent review covering the development of PET imaging agents for PgR [59].

Within the superfamily of nuclear hormone receptors [95], PgR is a member of the 3C subfamily, which includes AR (see Section 4 below), as well as the glucocorticoid receptor (GR) and the mineralocorticoid receptor (MR). Although less thoroughly investigated than the three receptors, ER, PgR, and AR, discussed here in detail, GR and MR certainly play roles in various cancers, including breast [89,96,97] and possibly prostate cancers. Hence, it is worth noting that some efforts have been made to develop imaging agents for these nuclear receptors: for GR [88,98,99,100,101] and for MR [88]. Limited in vivo studies using as endogenous targets for GR and MR, on liver and kidney of adrenalectomized adult male rats, respectively, were not promising [88]. Unlike ER, PgR, and AR, GR and MR need to respond physiologically to rapidly changing levels of their cognate corticosteroids, cortisol and aldosterone, so they tend not to retain their receptor-bound ligands as is needed for image contrast to develop. Thus, they are likely to be very challenging targets for PET imaging and will require particularly high-performing radiotracers.

## 4. FDHT, a PET Imaging Agent for Androgen Receptors in Prostate Cancers

### 4.1. A Prelude to In Vivo Imaging of AR: Optimizing the Binding Characteristics and Pharmacokinetic Properties of Radiotracers Leading to FDHT

Some early reports described radiobrominated and radioiodinated androgens for imaging AR in prostate cancer, but their AR binding was poor or not characterized, they were chemically or metabolically unstable, or they did not show clear evidence of AR-selective uptake in rodent prostate [102,103,104,105]. More recent work on radioiodinated androgens was more promising, and those with 7α-iodo or 17α-iodovinyl groups bound well to AR, although those lacking a C19 methyl group (4-estren-3-one core) begin to resemble synthetic progestins and risked having considerable affinity for PgR. In vivo biodistribution studies of uptake of these compounds in rat prostate were either limited or largely unpromising [24,64,66,68,106,107].

Because of our success with FES and the increasing availability of PET imaging, early on we focused our development of AR PET imaging agents on steroids labeled with fluorine-18. Prior to undertaking the preparation of candidate AR-targeting probes, we conducted a review of what was known in the literature about structure-activity relationships (SAR) of androgens [6]. (At the same time, we reviewed the SAR of progestins in preparation for our later investigations of ligands for PET imaging of the progesterone receptor (PgR) (see Section 3.1).) We supplemented what we learned from the literature on the SAR of AR ligands with some focused syntheses of fluorine-substituted analogs, the results of which were published in a review article [69].

**Figure 14:**
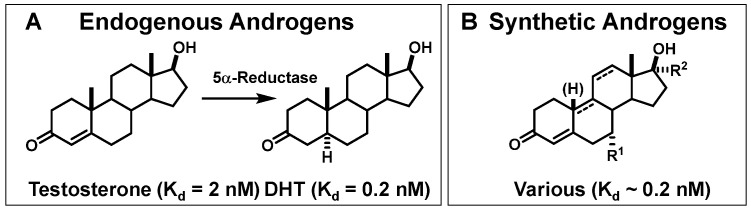
(**A**) Principal endogenous androgens and (**B**) high-affinity synthetic androgens.

As was the case with PgR, we noted that the AR binding affinity of testosterone, the principal circulating androgen, was not exceptionally high (Figure 14A). Compared to the subnanomolar affinity of estradiol for ER (K_d_ = 0.2 nM), the affinity of testosterone for AR was 10-fold lower (K_d_ = 2 nM), although the natural metabolite, 5α-dihydrotestosterone (DHT), generated in the prostate by the action of 5α-reductase, binds 10-fold better (K_d_ = 0.2 nM). In addition, many androgens are known to undergo very rapid metabolism, clearing at rates substantially greater than that of estrogens [6,69,108]. Considering both of these issues, we included in our studies some synthetic androgens known to have both very high binding affinity for AR and to be relatively resistant to metabolic clearance, such as mibolerone and methyltrienolone (Figure 14B).

**Figure 15:**
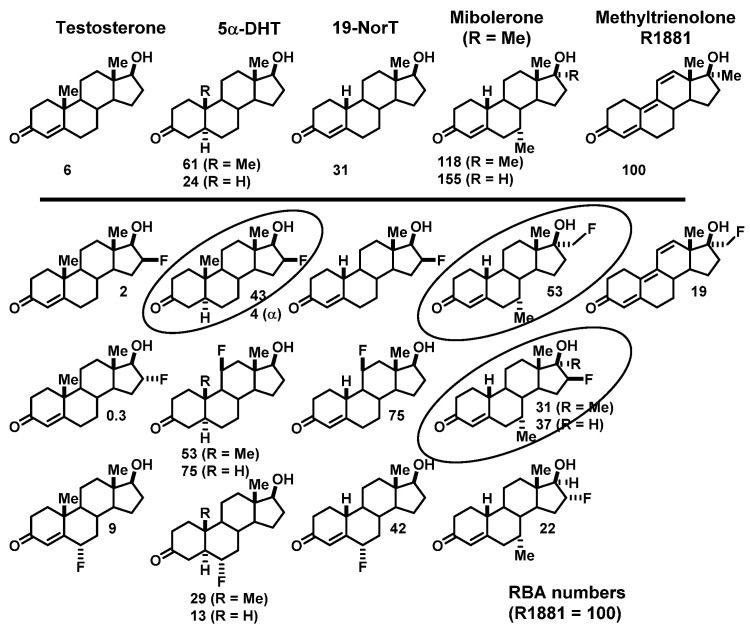
Five androgen receptor (AR) ligand groups (**top**) with fluorine-substituted analogs below. Numbers are AR binding affinities relative to methyltrienolone (R1881). The three circled structures were selected for further study.

We prepared a number of androgens (Figure 15, top) based on testosterone and four high affinity ligand series: 5α-dihydrotesterone (DHT), 19-nortestosterone, mibolerone, and methyltrienolone (the last, a compound developed by Roussel, also named R1881). As with the estrogens, binding affinities are given on a percent scale relative to that of the most commonly used radiotracer for competitive radiometric binding assays, methyltrienolone, which was assigned an RBA value of 100 (K_d_ = 0.2 nM) [6]. Both reduction of the 4,5-double bond in testosterone (which occurs endogenously by the action of 5α-dihydroreductase in the prostate) or removal of its 19 methyl group (as a synthetic analog) increased binding affinity by 5–10-fold. Strategic methyl substitution in the analogs, mibolerone and methyltrienolone, afforded both high binding affinity and was intended to provide relative resistance to metabolism, as did other structural alterations in methyltrienolone [6,69].

Below each of the five parental structures are a selection of fluorine-substituted analogs we investigated, showing their binding affinities relative to that of methyltrienolone (Figure 15, bottom). In most cases, introduction of the fluorine could be done conveniently late in the synthesis, by fluoride ion displacement of a triflate precursor [109,110], or in one case, a spirocyclic sulfate precursor [109,111]. The only one synthesized by a different route was 11β-fluoro-5α-dihydrotestosterone, in which fluorine was introduced by an unusual bromo-fluorination reaction on a Δ^9,(11)^ precursor, followed by rapid removal of a 9α-bromine by radical reduction with tributyltin hydride under forcing conditions [112]. Protecting groups that were sometimes needed could then be removed [109,111]. The fluorine-substitution and any needed deprotection reactions proceeded rapidly and under mild conditions, and was later readily adapted to radiolabeling with [^18^F]fluoride ion [109,111,112]. In most cases, fluorine substitution reduced binding affinity a few fold, and sometimes to a greater extent; nevertheless, several of the fluorine-substituted analogs still had subnanomolar binding affinities (RBA >20), and together with their low NSB values, their BSI values were quite reasonable.

Before studying the biodistribution of all of these analogs in a preclinical model, we carefully analyzed the level of AR in the prostate of adult male rats and the degree to which it was occupied by the normally high circulating levels of testosterone. We did this by comparing the effect of reducing endogenous testosterone levels by castration vs. by feedback inhibition from pretreatment with high doses of an estrogen (diethylstilbesterol, DES) on the biodistribution of [^3^H]R1881, [^3^H]testosterone, [^3^H]19-nortestosterone, [^3^H]DHT, and [^3^H]mibolerone. In all cases, we found robust and AR-selective uptake in the prostate, and although castration more markedly reduced AR occupancy by endogenous androgens, high-dose estrogen pretreatment was nearly as effective and proved much more convenient [113]. While some of these AR ligands (notably, R1881 and mibolerone) also have some affinity for PgR, the prostate uptake of these five tritiated test ligands was AR-specific because it was effectively blocked by simultaneous administration of a saturating dose of unlabeled DHT, but was unaffected by triamcinolone acetonide, which has high affinity for both PgR and GR [113].

When tested in the DES-pretreated adult rat model, most of our high affinity radiofluorinated compounds also showed marked uptake by the prostate that was effectively blocked by the AR ligand DHT, but not by triamcinolone acetonide [111,112,113,114]. At 2 h postinjection, prostate/muscle ratios for most compounds were typically 6–14, and were reduced by 60–80% after blocking with DHT; prostate/muscle ratios rose to as high as 25 at 4 h [17]. As typical for steroids, there was considerable activity in organs involved in metabolism and excretion (liver, kidney, and intestine) that was not blockable.

**Figure 16:**
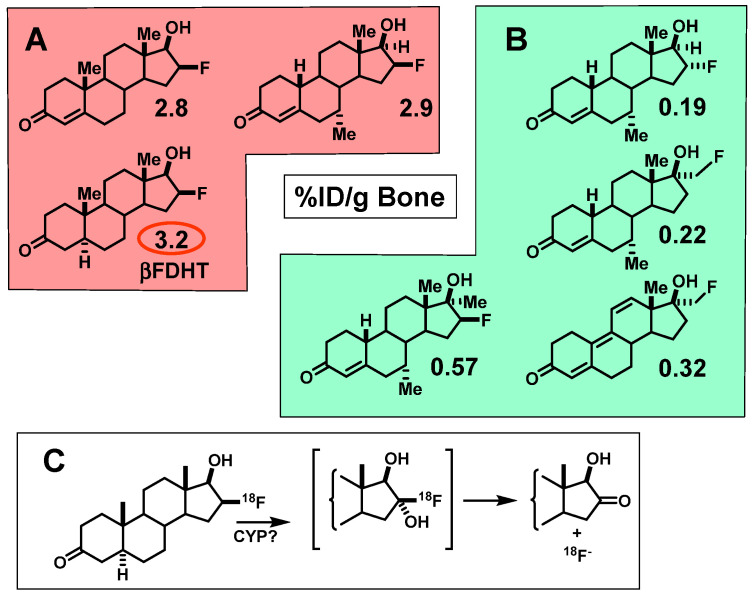
Metabolic defluorination of various fluorine-18 labeled AR ligands in rodent models. Numbers are the %ID/g bone activity. (**A, coral**) Those having an exposed 16α-hydrogen undergo extensive defluorination and have high bone uptake. (**B, teal**) Those lacking a 16α-hydrogen have less defluorination. (**C**) Proposed 16α-hydroxylation results in loss of F-18.

In contrast to the behavior of the F-18 labeled estrogens, we found that some radiofluorinated compounds underwent rapid defluorination, evident by activity uptake in bone that was not reduced by a blocking dose of unlabeled DHT (Figure 16) [111,112,113,114]. Defluorination was greatest with 16β-fluorine substitution, presumably due to an active 16α-hydroxylase activity. Fortunately, we soon found that this potentially undesirable pathway was not observed in biodistribution studies in nonhuman primates [17], and this was later confirmed in FDHT imaging studies in humans [3,115].

**Figure 17:**
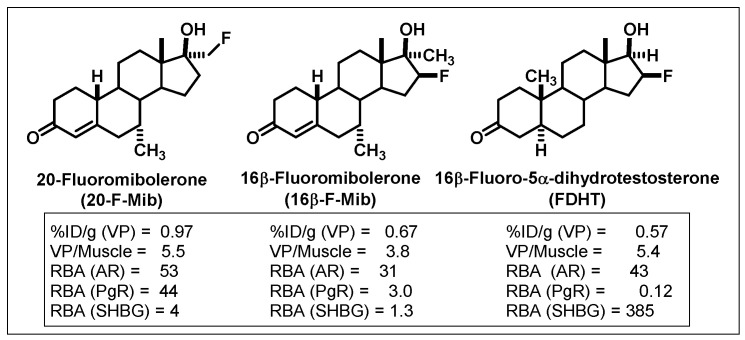
Three AR ligands evaluated in nonhuman primates. In androgen-suppressed adult male rats, %ID/g is at 1 h and ventral prostate (VP)/muscle ratio is at 4 h. RBA are relative to standards: for AR, R1881 = 100; for PgR, R5020 = 100; and for sex hormone binding globulin (SHBG), estradiol = 1.

We studied three of our promising compounds, 16β-[^18^F]fluoro-5α-dihydrotestosterone (FDHT), 16β-[^18^F]fluoro-mibolerone, and 21-[^18^F]fluoro-mibolerone (encircled in Figure 14), in a nonhuman primate model, a baboon [17]. All three compounds had shown essentially equivalent AR binding affinity and AR-specific uptake in the prostate of androgen-suppressed adult rats [111,114], but they had very different affinities for PgR (20-F-Mib was very high) and for the serum steroid carrier protein, sex steroid binding globulin (SHBG, FDHT was very high) (Figure 17). Quite to our surprise, FDHT gave clearly better and more selective uptake than the other two in baboon prostate [17]. We believe that this difference is due to the greater affinity that FDHT has for the blood steroid carrier protein, SHBG, than the mibolerone analogs [17,109,111]. SHBG is found in primates but not rodents. As we had noted during our development of PET probes for ER, some level of binding of FES to SHBG also seemed important for effective ER tracer distribution in vivo [18]. In addition, unlike the high bone activity found in rats due to metabolic defluorination (Figure 15), minimal bone activity was found in baboons [17]. Hence, FDHT was chosen as a compound for further studies in humans. The first FDHT-PET imaging of AR in men with advanced prostate cancer was published together with my colleagues at Washington University Medical School [3] and nearly simultaneously by investigators at Sloan Kettering Cancer Center [115]. In both studies, clear FDHT uptake was evident in numerous lesions, with uptake being blocked following treatment with AR antagonists. Further discussion of FDHT-PET imaging in prostate cancer and its potential utility in management of this disease are presented in Section 5.3.

### 4.2. Opportunities and Efforts to Improve the Imaging Characteristics of FDHT in Prostate Cancer

In early FDHT-PET imaging studies, a circulating lipophilic but somewhat more polar radiolabeled metabolite was noted [115], and although it did not bind to AR and was not taken up by prostate cancer lesions, it made accurate measurement of blood levels of FDHT for pharmacokinetic modeling more difficult [116]. Its almost immediate formation in blood suggests that rapid metabolism may also be limiting AR-mediated uptake of FDHT. Thus, it led us to consider further potential refinements of PET imaging agents for AR.

A-ring saturated androgens like FDHT are known to undergo rapid reduction by 3α-hydroxysteroid dehydrogenases (3α-HSDs), converting the 3-ketone to the 3α-hydroxy analog, which has very poor AR binding affinity [6,69,108]. Though it has not been unequivocally verified, the labeled metabolite noted in the Sloan-Kettering studies [115,116] was likely the 3α-hydroxy analog of FDHT, 16β-[^18^F]fluoroandrostane-3α,17β-diol.

**Figure 18:**
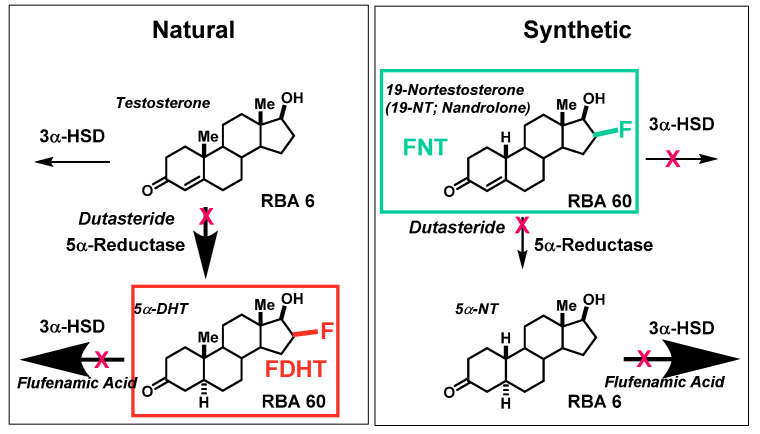
Design consideration to increase metabolic stability and extend circulation time of an AR PET probe. Boldness of arrows indicates relative rates. Red X’s show sites of metabolism inhibition.

The known routes of metabolism of different androgens by 3α-HSD and 5α-reductase (5α-R) are displayed in Figure 18, and this scheme illustrates for different steroids, the contrasting preference or resistance for these two pathways [69]. The 4,5-double bond in both testosterone and 19-nortestosterone makes them poor substrates for the 3α-HSD, which would make them relatively resistant to inactivation by this pathway. Testosterone, however, is a good substrate for 5α-R, which would eliminate the protecting 4,5-double bond. This would generate the higher affinity for 5α-dihydrotestosterone, which would be rapidly metabolized by 3α-HSDs; by contrast, 19-nortestosterones are poor 5α-R substrates. The boldness of arrows in the scheme illustrates the relative rates of these two potential routes of metabolism, and one can see that FDHT, as a dihydrotestosterone analog, is at risk of rapid inactivation by 3α-HSD activity, whereas a corresponding 19-nortestosterone tracer, which is a poor substrate for both 3α-HSD and 5α-R, is likely to have more extended blood residence and higher AR-mediated target uptake. Thus, more in-depth investigations of F-18 labeled 19-nortestosterone and methyltrienolone analogs might be productive. Inhibitors of both 3α-dehydrogenases (flufenamic acid and others) [117] and 5α-reductases (dutosteride) [118] are available, and pretreatment with these drugs might slow the clearance of these types of tracers and enhance their uptake efficiency and selectivity and reduce non-AR binding circulating metabolites. These ideas for enhanced AR PET imaging agent design and drug treatment paradigms are open to future investigations.

**Figure 19:**
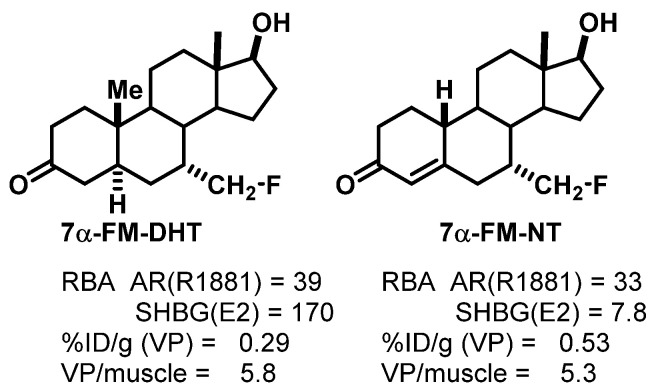
Matched set of fluorine-18 labeled AR PET probes with equivalent AR binding and uptake efficiency and selectivity, but very different affinities for SHBG.

Another opportunity that might merit further investigation would be to better define the role of SHBG in the efficiency and selectivity of imaging AR in prostate cancers, which proved to be a challenging problem to address in preclinical models [119]. As a prelude to such an investigation, we prepared two AR ligands that were closely matched structurally and had very similar AR binding affinities, but very different SHBG binding affinities (Figure 19). As we had hoped, in rats, which lack SHBG, these two showed very similar uptake in the prostate. The definitive experiments to test the role of SHBG would involve comparing these two in nonhuman primates, as we had done earlier with rather different compounds [17], or better yet, in men with prostate cancers.

There are also a whole host of nonsteroidal ligands for AR, developed for various endocrine therapeutic purposes, such as antiandrogens to treat prostate cancer (enzalutamide, apalutamide, darolutamide, and earlier flutamide and bicalutamide) and selective androgen receptor modulators (SARMs) for treating men with hypogonadal conditions (many compounds from GTx) [120]. The thiohydantoin core of most of these compounds was actually first described many years ago [121]. Because some of these have high AR binding affinity and are small, relatively polar compounds, we radiolabeled a few of them with fluorine-18 or bromine-76; however, none of them showed AR-selective prostate uptake in our preclinical model [122,123,124]. Note is made of another recent review covering the development of PET imaging agents for AR [59].

## 5. Considerations for How PET Imaging of Steroid Receptors Might Best be Used to Improve the Management of Breast and Prostate Cancers

It might seem axiomatic that PET imaging of ER with FES and PgR with FFNP in breast cancers, and AR with FDHT in prostate cancers, would be of use in detecting and staging disease and as prognostic and predictive factors in guiding optimal treatment for women and men with these cancers. An evaluation of their clinical utility, however, requires deeper analysis of the distinct roles played by these three receptors in their associated cancers, as well as consideration of the extent to which the information they provide goes beyond that can be obtained from other more easily available imaging modalities and from biomarker analyses on tumor biopsy samples. These issues will be discussed in the three sections that follow. Irrespective of their use in precision medicine to guide optimal endocrine therapy for individual patients, however, PET imaging of ER [125,126,127] and AR [128,129] in tumors plays a key role in guiding optimal dose selection during the clinical development of new receptor antagonists through pharmacodynamic or target engagement studies.

### 5.1. Utility of FES-PET Imaging of ER in Breast Cancers for Guiding Endocrine Therapy Selection

As early as the late 1890s, long before ER and its activating ligand estradiol were identified, some women with breast cancer were found to respond—often dramatically—to surgical removal of their ovaries. This discovery by Sir George Beatson in 1896 is considered the first example of targeted endocrine therapy for cancer [130,131]. It soon became apparent that surgical endocrine ablation was effective in only about one-third of breast cancer patients [132]. Thus, even a century ago, it was clear that a means for predicting response to endocrine therapy was needed to spare many women with breast cancer from the morbidity of a surgery that was likely to be ineffective.

Of course, we now know that ER is a major driver of proliferation in many breast cancers and is the target for endocrine therapies, such as aromatase inhibitors and antiestrogens. In fact, about 70% of breast cancers have detectable levels of ER and are termed ER-positive, but only about half of ER-positive disease actually responds to endocrine therapy, with response rates being lower in more advanced or recurrent disease. Thus, irrespective of how ER assays are being done, positive predictive values (PPVs) are very low, only around 50%. (Negative predictive values (NPVs) are much higher.) Clearly, improved methods are still needed to predict whether ER-positive cancers will respond to endocrine therapies, which have relatively mild side effects, or whether more aggressive radiation or chemotherapies are warranted, despite their greater morbidities [133,134].

Earlier radiometric assays measured ER levels by the direct binding of [^3^H]estradiol, ex vivo on extracts of fresh or frozen tumor samples; current ER IHC assays measure an antigenic signal from ER on formalin-fixed, paraffin -imbedded (FFPE) sections from fine-needle biopsies and are graded on scales that consider staining intensity and distribution of stained cells [135,136]. By contrast, FES-PET imaging of breast tumors provides a more direct measurement of ER, based on its ligand-binding *function* that is assessed in vivo and in situ, and noninvasively (recently extensively reviewed [133,137]). There are sensitivity limits to FES-PET, and imaging of lesions in liver and near the colon is difficult due to high background from FES metabolism and hepatobiliary elimination, yet multiple tumor sites can be evaluated by FES-PET without patient discomfort, even those that are inaccessible (e.g., brain), are challenging to reach by needle biopsy, or require tissue processing (bone) [138,139,140]. ER levels in recurrent disease can also differ from those determined in the primary tumor at an earlier time [137,141], and FES-PET can provide a measurement at the time of treatment decision. PET also provides an image of the whole tumor and avoids the risk of sampling error from fine needle biopsies due to intratumoral heterogeneity in ER levels [142].

In early FES-PET studies, we showed FES-specific uptake in primary breast tumors correlated quantitatively with ER levels determined subsequently by radioligand binding assays on biopsy samples [51]. FES uptake in metastatic disease also correlated with earlier radiometric ER determinations from the primary tumor [143]. Notably, there was no correlation between tumor uptake by FES-PET and [^18^F]fluorodeoxyglucose (FDG)-PET [144]; only FES-PET was predictive of benefit from endocrine therapy [145]. In advanced breast cancers, FES-PET provided more accurate prognoses and prediction of endocrine therapy success [146], adding value over that from ER IHC assays and FDG-PET images [147], particularly in patients otherwise difficult to evaluate [133,147,148,149,150,151,152].

A number of active clinical trials (some multicenter) continue to investigate the predictive value of FES-PET, often together with FDG-PET or other targeted imaging agents, or to assess its ability to identify metastases and intrapatient heterogeneity ((NCT01957332), (NCT02398773), (NCT03726931), and (EUDRACT 2013-000-287-29)). Suggestions for best practices in the clinical use of FES-PET have been published [153], as has a simulation of the cost effectiveness of FES-PET vs. conventional work-up of patients with metastatic breast cancer [154]. Zionexa, a French radiopharmaceutical company, has recently obtained approval for the clinical use of FES in France and the US. FES, termed “Cerianna” by Zionexa and registered as EstroTep^®^, is now available for clinical use in the US and should be widely available commercially through an agreement between Zionexa and PETNET. A very recent publication in *The Oncologist* provides a comprehensive analysis of the clinical value of FES-PET imaging in breast cancer [155]. Because of the high negative predictive value of FES-PET, it is likely to be particularly useful in predicting the absence of significant ER levels in metastatic disease, although its utility in providing more definitive prognostic and predictive information about other stages of disease are likely to be identified.

The value of FES-PET imaging needs to be considered within the larger landscape of other approaches to predict benefit from endocrine therapy in breast cancer. Much current research seeks to predict endocrine therapy response in advanced breast cancer using various “-omics” (genomic, cistromic, transcriptomic, proteomic, and metabolomic) databases derived from analyses of tumor biopsy samples, as well as liquid biopsies of circulating tumor cells and circulating tumor DNA in metastatic breast cancer. “Signatures” derived statistically from these databases are used to define molecular pathological subtypes that are then correlated with disease prognosis and prediction of therapy response. A good example comes from a recent clinical trial (TAILORx) showing that a 21-gene profile (Oncotype-Dx) effectively identified patients with early stage ER-positive, node-negative disease who are very likely to benefit from endocrine therapies alone [156]. In advanced recurrent and metastatic ER-positive disease, endocrine therapy response rates are much lower and depend on prior therapies [157,158], and currently, there are no -omics assays that robustly predict benefit from endocrine-only therapy. It is in this context that the value of FES-PET is most likely to add predictive value to the benefit from continued endocrine therapies. In a yet larger context, PET imaging presents an interesting alternative approach of directly accessing whether ER in tumors remains functional through the use of “hormone challenge tests.” Such PET imaging-based challenge tests are discussed below in a broader context (Section 5.2.).

### 5.2. Clinical Utility of FFNP-PET Imaging of PgR in Breast Cancers: PET Imaging-Based Hormone-Challenge Tests

Clinical assays of ER in breast cancers have good negative predictive values (NPVs), but much poorer positive predictive values (PPVs); it seems, in fact, that ER can be present, but somehow incapable of mediating endocrine therapy. Because the PgR gene is highly regulated by estrogen action through ER [159,160,161], as early as the 1970s, the measurement of breast tumor PgR levels was proposed as a way to improve the PPV of receptor assays for endocrine therapy benefit [162,163]: an ER-positive tumor that was also PgR-positive would indicate that ER was functional and would respond to endocrine therapy, whereas an ER-positive/PgR-negative tumor would not respond, because the ER, somehow, was nonfunctional. Consequently, breast cancer biopsies have been routinely assayed for both ER and PgR for many years. The logical and attractive rationale notwithstanding, however, PgR assays have not had a transformative effect on the predictive value of an ER-positive result, particular in advanced disease. In fact, the value of PgR measurements remains an area of active disagreement in recommendations for best clinical practice in breast cancer [164,165,166,167,168,169].

**Figure 20:**
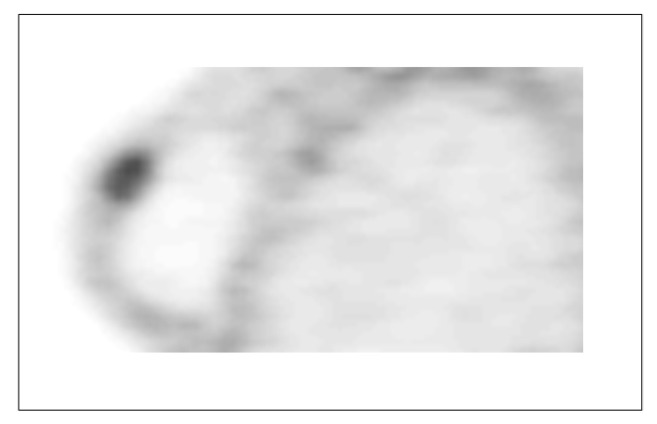
FFNP-PET sagittal image of breast cancer in woman with ER+/PgR+/HER2-disease. (Image provided by Drs. Farrokh Dehdashti and Barry Siegel, Washington University Medical School.)

As most breast cancers are found in older, postmenopausal women, a reasonable but not widely considered explanation is it that PgR assays in these women are compromised because their low endogenous levels of estradiol are insufficient to elevate PgR levels, even when ER is active. In our initial clinical study of FFNP-PET in breast cancer patients (all of whom were postmenopausal) (NCT00968409), we found that FFNP uptake was greater in PgR-positive than PgR-negative breast cancers (Figure 20) and the average SUV values were separated; uptake values, however, were neither very high nor markedly separated [4], very likely because of the low levels of circulating estradiol in these women. (There are other ongoing clinical trials using FFNP-PET on invasive breast cancer at the University of Wisconsin (NCT03212170).)

Considering these issues, we hypothesized that the value of PgR as a biomarker for functional ER would be clearer if it were used as a dynamic biomarker, by FFNP-PET imaging of PgR levels in tumors before and then shortly after a brief, stimulating dose of estradiol: if tumor ER was functional, we expected to see an increase in FFNP-PET SUV, whereas the absence of an increase would suggest that ER was nonfunctional. We demonstrated the validity of such an PgR based “estrogen-challenge test” through proof-of-principle preclinical studies in syngeneic murine mammary cancer models derived from STAT1 knockout mice [170]; by serial Western blot analyses and FFNP-PET imaging, PgR levels decreased with estradiol deprivation and increased after a 1-day estradiol stimulation in the ER-positive and responsive SSM3 tumors, whereas there were no apparent changes in the ER-positive nonresponsive SSM2 tumor system [84,85]. A more recent study has tracked the time course of PgR level increase in human breast cancer T47D xenografts in mice by biodistribution, with levels continuing to rise even after 2-days of estrogen treatment [171]. While treating women with ER-positive breast cancer with estradiol, an ER agonist, would seem counterintuitive, high-dose estrogen (originally diethylstilbestrol) was actually the first form of endocrine therapy [172,173]. Response rates are equivalent to those with antiestrogens, but because of side effects, high-dose estrogen therapy has been replaced by antiestrogens [174], although it is still used in certain advanced disease [175,176].

In a clinical study at Washington University that was just completed (NCT02455453), we used FFNP-PET to monitor the change in PgR after a 1-day estradiol challenge in 43 women with ER-positive advanced breast cancer that had failed on various prior endocrine therapies and chemotherapies, but were still candidates for treatment with the powerful antiestrogen fulvestrant or other endocrine therapies, often combined with CDK4,6 inhibitors. The value of the change in FFNP-PET SUV before and after the 1-day treatment with estradiol in predicting clinical benefit is being compared with baseline FFNP SUV and PgR by IHC. The results from this trial will be reported shortly.

It is worth considering our current use of a hormone-challenge paradigm in PET imaging in breast cancer within the broader landscape of endocrinology (for a more extensive review, see reference [47]). Breast cancer is an endocrine-driven disease, and challenge tests of various types are commonly used in endocrinology, examples being the glucose challenge test for diabetes and the dexamethasone suppression test to assess adrenal gland function. In this regard, I was intrigued by the clinical observation of a “tamoxifen flare” [177]. This was a transient exacerbation of clinical pain symptoms experienced in some breast cancer patients about 1–2 weeks after starting on tamoxifen therapy that would then subside as patients began to benefit from the continued endocrine therapy. This tamoxifen flare was ascribed to a transient agonist effect of tamoxifen early in the course of treatment as therapeutic levels were building up [178]. Later, it was demonstrated that very low levels of tamoxifen will actually stimulate MCF-7 breast cancer cell growth [179], although higher levels are inhibitory [180]. Clinical tamoxifen flare was not very useful as a predictor of benefit from tamoxifen, because responders without bone metastases rarely experienced a flare in pain, and some nonresponders experienced increasing pain due to disease progression. Thus, pain was not the proper parameter to use to monitor this transient stimulation of tumors in which ER was functional, a response that actually represented an early response to what would eventually be a beneficial outcome.

FDG-PET imaging is widely used in cancer for assessing the extent of disease, but it is of less value in detecting “indolent disease,” because the Warburg effect develops when cancer cells are avidly proliferating. If tamoxifen flare was the result of a transient stimulation of the tumor by the agonistic effect of low-dose tamoxifen that cannot be reliably detected by pain symptoms, perhaps the flare could be more readily detected by changes in tumor metabolism monitored by two FDG-PET images, one at baseline and one after 1–2 weeks of tamoxifen treatment, the time of maximum clinical tamoxifen flare development. Thus, we added to our early clinical studies of FES-PET imaging in ER-positive breast cancer two FDG-PET images, i.e., women received both FES-PET and FDG-PET at baseline and then a second FDG-PET image at 7–10 days, after tamoxifen therapy begin. While the FES-PET image was more predictive of therapy benefit than the results of the clinical assay of ER, the change in FDG-PET image was even more predictive [144,181,182,183]. When aromatase inhibitors replaced tamoxifen as preferred endocrine therapy, we modified the hormone challenge paradigm to be just 1 day of estradiol prior to the onset of aromatase inhibitor treatment, because the therapy agent would no longer cause transient stimulation of responsive tumors [184]. Again, the change in FDG-PET SUV from this estradiol challenge test of metabolic flare proved to be very predictive, even in women with advanced breast cancers, many of whom had received prior therapies of various kinds.

Though predictive, the changes we saw in FDG-PET were relatively modest, with many values clustering around the positive–negative cut off value. Our recent clinical study of the changes in PgR measured by FFNP-PET in advanced breast cancer, uses the same 1-day estradiol challenge paradigm (NCT02455453). By monitoring PgR level rather than FDG uptake rate, we are hoping to find a larger separation between responders and nonresponders in the change of FFNP SUV than what we had found with FDG-SUV. In the aforementioned preclinical trial with FFNP in a mouse mammary tumor model, we had found the changes in FFNP-SUV to be much larger than the changes in FDG-SUV [84,85].

### 5.3. Clinical Studies of FDHT-PET in Prostate Cancer

**Figure 21:**
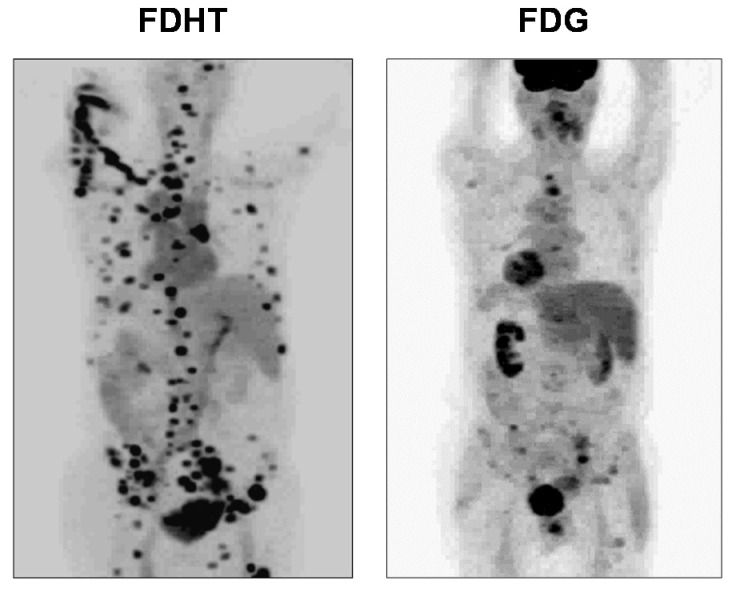
FDHT-PET and FDG-PET image of prostate cancer in an individual with FDHT-avid disease. (Images provided by Dr. Steven Larson, Memorial Sloan Kettering Cancer Center.)

PET imaging of AR in men with advanced prostate cancer was published in 2004 and 2005 by me and my colleagues at Washington University Medical School in St. Louis [3] and by investigators at Memorial Sloan Kettering Cancer Center in New York (Figure 21) [115]. Clear FDHT uptake was evident in numerous lesions, with uptake being blocked following treatment with AR antagonists. Subsequent studies made comparisons between FDHT and FDG images in the same patient to characterize lesion heterogeneity, and comparative images in a series of patients showed both concordance (i.e., high or low uptake of both PET tracers) and discordance (FDHT high, FDG low, and the reverse) [185]. Some prognostic trends were correlated with these four distinct patterns of tracer uptake, with survival being greatest when FDG and FDHT images were concordant and lowest when FDHT uptake by lesions was very low. Survival was also inversely related to total lesion count and intrapatient heterogeneity; notably, PSA levels had little prognostic value [185]. Because often many more lesions are seen by PET imaging than can be biopsied, the imaging results can direct tissue sampling to the sites of possibly greatest concern (e.g., high metabolic activity (high FDG PET) and low AR responsiveness (low FDHT PET)) [185].

The predictive and prognostic role of AR in prostate cancer is rather different than that of ER in breast cancer. With ER-positive breast cancers, endocrine therapies often become less effective in advanced disease; so PET imaging of ER with FES and ER functional status by serial FDG- or FFNP-PET with an estradiol challenge can provide additional predictive value for benefit from continued endocrine therapies. By contrast, as long as AR is present in prostate cancer, it typically remains an effective target for hormone-directed therapies, even in advanced stages of castration-resistant prostate cancer (CRPC), although increasingly powerful endocrine strategies are needed [186]. The conversion from CRPC to neuroendocrine-type cancer typically involves loss of both AR and responsiveness to AR-targeted therapies [187]; so, FDHT-PET might prove useful in identifying this clinically significant progression by imaging.

More recently, extensive development of PET tracers for prostate-specific membrane antigen (PSMA), which is widely expressed in prostate cancer, has produced agents of superb selectivity for detection of lesions; these are likely to play a major role in PET imaging of prostate cancer [188,189]. Further investigations should help to clarify the relative value of FDHT-PET vs. PSMA-PET imaging in guiding therapies for prostate cancer, though it is likely that both will have distinct roles to play, as will FDG-PET [185,188,189]. PSMA also appears to be an effective target for radiotherapy agents that show efficient update and very prolonged retention in lesions; localization in cell nuclear structures make possible selective radioablation [190], even using Auger electron-emitting radionuclides [191].

Nevertheless, FDHT has proven to have enduring value in pharmacodynamics or receptor occupancy studies that provide valuable guidance for dose selection in clinical trials for the development of new antiandrogens. Such studies have been used for the development of enzalutamide (Xtandi) [129] and apalutamide (Erleada) [128].

## 6. Closing Comments

The capability of PET as a modality for imaging *function* is unparalleled; this was articulated in an insightful comment made to me by Henry Wagner, a grandfather of the field, who said, “PET can image function better than any other mode of clinical imaging!” [192]. The prognostic and predictive value of PET imaging of ER by FES, PgR by FFNP, and AR by FDHT compare very favorably in many respects with the results of IHC assays. PET imaging, however, also provides several advantages by being noninvasive and assessing multiple sites and by measuring in vivo and in situ the capacity of the receptor to bind its cognate ligand; this assessment of the binding function of these receptors, not an antigenic feature, is of immense value. Beyond imaging receptor-binding function, the use of serial PET imaging in a hormone-challenge paradigm, as we have done with FDG-PET to assess changes in tumor metabolism and FFNP-PET to assess changes in PgR gene expression, goes beyond hormone binding function; it rapidly evaluates the functional status of ER regulation of cellular activities in cancer that are associated with response to endocrine therapy. There is no reason that hormone challenge tests based on imaging other therapy-responsive functions in cancer could not also be developed, such as ones that would use PET probes for different cellular activities such as proliferation, hypoxia, and protein and DNA synthesis. Hence, the use of PET imaging of steroid receptors enables observation of the extent of cancer metastasis and predicts responsiveness to endocrine therapies. Its use in assessing new aspects of tumor clinical status noninvasively suggests a robust future for PET imaging in providing meaningful benefit for patients with breast and prostate cancers.
